# Solvation, Surface Propensity, and Chemical Reactions of Solutes at Atmospheric Liquid–Vapor Interfaces

**DOI:** 10.1021/acs.accounts.2c00604

**Published:** 2022-12-06

**Authors:** Markus Ammann, Luca Artiglia

**Affiliations:** Laboratory of Environmental Chemistry, Paul Scherrer Institute, 5232 Villigen, Switzerland

## Abstract

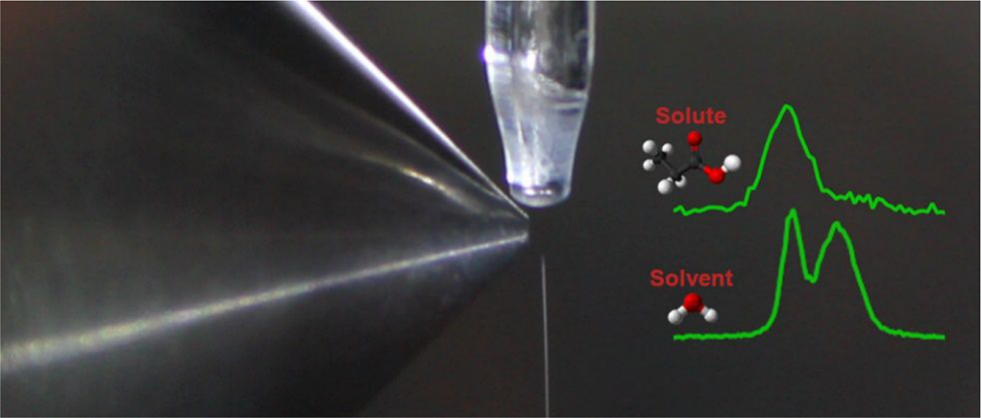

Liquids are of overarching importance for the atmosphere, as 72% of the Earth’s surface is covered by oceans, a large number of liquid aerosol particles fill the air, and clouds hold a tiny but critical fraction of Earth’s water in the air to influence our climate and hydrology, enabling the lives of humans and ecosystems. The surfaces of these liquids provide the interface for the transfer of gases, for nucleation processes, and for catalyzing important chemical reactions. Coupling a range of spectroscopic tools to liquid microjets has become an important approach to better understanding dynamics, structure, and chemistry at liquid interfaces. Liquid microjets offer stability in vacuum and ambient pressure environments, thus also allowing X-ray photoelectron spectroscopy (XPS) with manageable efforts in terms of differential pumping. Liquid microjets are operated at speeds sufficient to allow for a locally equilibrated surface in terms of water dynamics and solute surface partitioning. XPS is based on the emission of core-level electrons, the binding energy of which is selective for the element and its chemical environment. Inelastic scattering of electrons establishes the probing depth of XPS in the nanometer range and thus its surface sensitivity.

In this Account, we focus on aqueous solutions relevant to the surface of oceans, aqueous aerosols, or cloudwater. We are interested in understanding solvation and acid dissociation at the interface, interfacial aspects of reactions with gas-phase reactants, and the interplay of ions with organic molecules at the interface. The strategy is to obtain a link between the molecular-level picture and macroscopic properties and reactivity in the atmospheric context.

We show consistency between surface tension and XPS for a range of surface-active organic species as an important proof for interrogating an equilibrated liquid surface. Measurements with organic acids and amines offer important insight into the question of apparent acidity or basicity at the interface. Liquid microjet XPS has settled the debate of the surface enhancement of halide ions, shown using the example of bromide and its oxidation products. Despite the absence of a strong enhancement for the bromide ion, its rate of oxidation by ozone is surface catalyzed through the stabilization of the bromide ozonide intermediate at the interface. In another reaction system, the one between Fe^2+^ and H_2_O_2_, a similar intermediate in the form of highly valent iron species could not be detected by XPS under the experimental conditions employed, shedding light on the abundance of this intermediate in the environment but also on the constraints within which surface species can be detected. Emphasizing the importance of electrostatic effects, we show how a cationic surfactant attracts charged bromide anions to the interface, accompanied by enhanced oxidation rates by ozone, overriding the role of surfactants as a barrier for the access of gas-phase reactants. The reactivity and structure at interfaces thus result from a subtle balance between hygroscopic and hydrophobic interactions, electrostatic effects, and the structural properties of both liquids and solutes.

## Key References

- LeeM.-T.; OrlandoF.; ArtigliaL.; ChenS.; AmmannM.Chemical Composition and Properties of the Liquid–Vapor Interface of Aqueous C1 to C4 Monofunctional Acid and Alcohol Solutions. J. Phys. Chem. A2016, 120, 9749–975810.1021/acs.jpca.6b0926127973794.^1^*This work establishes the correlation between XPS and surface tension for carboxylic acid, carboxylate and alcohol solutions, which is an important proof for interrogating an equilibrated liquid surface in liquid jet XPS experiments.*
- GladichI.; ChenS.; VazdarM.; BouclyA.; YangH.; AmmannM.; ArtigliaL.Surface Propensity of Aqueous Atmospheric Bromine at the Liquid–Gas Interface. J. Phys. Chem. Lett.2020, 11, 3422–342910.1021/acs.jpclett.0c0063332283032.^2^*This combination of liquid jet XPS and theoretical calculations establishes that bromide ions are not enhanced at the aqueous solution–air interface as suggested earlier. In turn, hypobromous acid turns out to be surface-active.*
- ArtigliaL.; EdebeliJ.; OrlandoF.; ChenS.; LeeM.-T.; Corral ArroyoP.; GilgenA.; Bartels-RauschT.; KleibertA.; VazdarM.; Andres CarignanoM.; FranciscoJ. S.; ShepsonP. B.; GladichI.; AmmannM.A surface-stabilized ozonide triggers bromide oxidation at the aqueous solution-vapour interface. Nat. Commun.2017, 8, 70010.1038/s41467-017-00823-x28951540PMC5615067.^3^*The surface-active bromide ozonide intermediate is driving the surface reaction of ozone with bromide in aqueous solution as seen by liquid jet XPS and theoretical and kinetic investigations.*
- GladichI.; ChenS.; YangH.; BouclyA.; WinterB.; van BokhovenJ. A.; AmmannM.; ArtigliaL.Liquid–Gas Interface of Iron Aqueous Solutions and Fenton Reagents. J. Phys. Chem. Lett.2022, 13, 2994–300110.1021/acs.jpclett.2c0038035344351.^4^*Fe*^*2+*^*and Fe*^*3+*^*ions form slightly distorted octahedral and octahedral aquo complexes, respectively. No sign of highly valent iron species is detected upon dosing gaseous H*_2_*O*_2_*at the liquid interface of an Fe*^*2+*^*solution.*

## Liquids in the Atmospheric Environment

Liquids based on water as a solvent are of overarching importance in the atmosphere. A large number of liquid aerosol particles float in the air,^5^ and clouds hold a tiny but critical fraction of Earth’s liquid water to influence our climate and hydrology.^6^ The surfaces of these liquids provide the interface to transfer important gases,^7^ to drive nucleation processes,^8^ and to catalyze important chemical reactions.^9^ An important fraction of atmospheric aqueous solutions are derived from seawater.^10^ These aqueous solutions contain halide salt ions but also a variable fraction of organic material from marine biota or after acquiring soluble organic species in the atmosphere.^11^ The oxidation of chloride, bromide, and iodide leads to gas-phase species, which significantly affect the ozone budget.^12,13^

Another important aerosol type is mineral dust.^14^ In the atmosphere, these particles undergo chemical processing by acidic gases, which leads to the formation of a liquid phase surrounding them^15^ and to the dissolution of some components, including iron.^16^ Dissolved iron also originates from other, anthropogenic particle sources, enters all environmental watersheds,^17^ and is globally relevant as a nutrient.^18^ Dissolved iron forms complexes, often with organic ligands, which are an important photolytic radical source in the atmosphere,^19^ affecting the lifetime of organic species, the burden of particulate matter, and its health impacts.^20^

A plethora of other solutes, such as nitrate, ammonium, sulfate, and organic solutes, are present in atmospheric aerosol and cloudwater^5^ but are not addressed in this Account. Liquid interfaces are also relevant in modern chemistry, where water surfaces enable or accelerate reactions otherwise inefficient in the aqueous bulk phase.^21^ Therefore, understanding the dynamics, structure, and chemistry at liquid interfaces is of tremendous and transdisciplinary importance. Aqueous solutions have been studied by means of structure-sensitive neutron and X-ray scattering^22^ and by IR and Raman spectroscopy to understand the local structure and chemistry.^23^ Due to its inherent sensitivity to the asymmetry of interfaces, nonlinear vibrational spectroscopy is particularly useful for characterizing the interfaces of aqueous electrolyte solutions,^24^ of surfactant-coated solutions,^25^ of water interacting with proteins,^26^ and of water in interaction with mineral surfaces.^27^ Here, we focus on X-ray photoelectron spectroscopy due to the combination of surface sensitivity and chemical selectivity.

## Liquid Microjet-Based X-ray Photoelectron Spectroscopy

Liquid microjets have become an increasingly popular method of investigating liquid–vapor interfaces,^28^ especially in combination with X-ray photoelectron spectroscopy.^29^ Liquid microjets are operated with 10 to 100 μm diameter at a typical speed of 100 m/s, leading to laminar filaments a few millimeter in length or 10 μs of traveling time to the point of detection. This time is sufficient to allow the establishment of a locally equilibrated surface in terms of water dynamics and solute surface partitioning.^30^ On local scales, the water evaporation rate in vacuum is slow in comparison to the hydrogen bond exchange dynamics.^31^ In turn, evaporative cooling is limited to very few degrees Kelvin during the 10 μs travel time.^32^

X-ray photoelectron spectroscopy (XPS)^33^ is based on the excitation of core or valence electrons with X-rays and the detection of the kinetic energy (KE) spectrum of photoelectrons. Photoelectron peaks at the KE corresponding to the difference between the photon energy and the binding energy (BE) of the electronic level provide information on the precise state of the electronic level and thus on the local environment of the atom involved. This provides chemical selectivity for the element or functional group, oxidation states, and degree of solvation or dissociation. The strong inelastic scattering of electrons in condensed matter drives the probing depth of the method through the KE and thus the surface sensitivity. The contribution of an atom to the photoemission peak intensity decreases exponentially with depth, with 80% of the signal from atoms within the top layer of thickness equal to the inelastic mean free path (IMFP), which for water ranges between 1 and 3 nm for KE between 100 and 1000 eV.^34^ Using a tunable synchrotron X-ray source allows the variation of the KE and thus the probing depth and allows the retrieval of the differences between the chemical composition at the surface and in the bulk. The process of filling the core hole generated by X-ray excitation with a valence electron followed by the emission of another valence electron is referred to as the Auger process. The yield of Auger electrons can be used to obtain surface-sensitive X-ray absorption spectra (XAS).^29a^ The excitation of core electrons into unoccupied molecular orbitals below the ionization edge leads to sharp resonances in XAS referred to as near-edge X-ray absorption fine structure (NEXAFS), which provides valuable information about the local molecular environment and thus complementary information to XPS.^29a^

XPS has been performed on liquids ever since the method was established,^35^ historically with low vapor pressure liquids^36^ and later with aqueous solutions of high ionic strength.^37^ The relatively small size of the liquid filament in microjet applications helps to reduce the efforts for differential pumping toward the electron detector (that needs to remain under ultrahigh vacuum). A major feature of liquid microjet XPS is the permanently renewed surface that avoids beam damage, with the typical velocities fast enough that radicals that are produced remain at sufficiently low concentration so as not to significantly affect any of the species in the solution.^3,30,38^

We have built an endstation for the Swiss Light Source (SLS),^39^ which features a differentially pumped electrostatic prelens to focus photoelectrons into the hemispheric analyzer. This allows the variation of the background pressure in the sample region from around 10^–4^ mbar to a few mbar. Indeed, as a side opportunity of this feature, we have demonstrated that the elemental density profiles as seen by KE-dependent XPS do not depend on whether the liquid jet is operated in vacuum or at equilibrated water vapor pressure for a K_2_CO_3_ solution.^40^ Beyond the capability to probe the liquid–vapor interface at both low and equilibrated pressure, the mbar range is essential for studying the liquid–vapor interface in the presence of carrier gases containing trace gases.

## Interfacial Solvation, Abundance, and Orientation of Organic Acids

**Figure 1:**
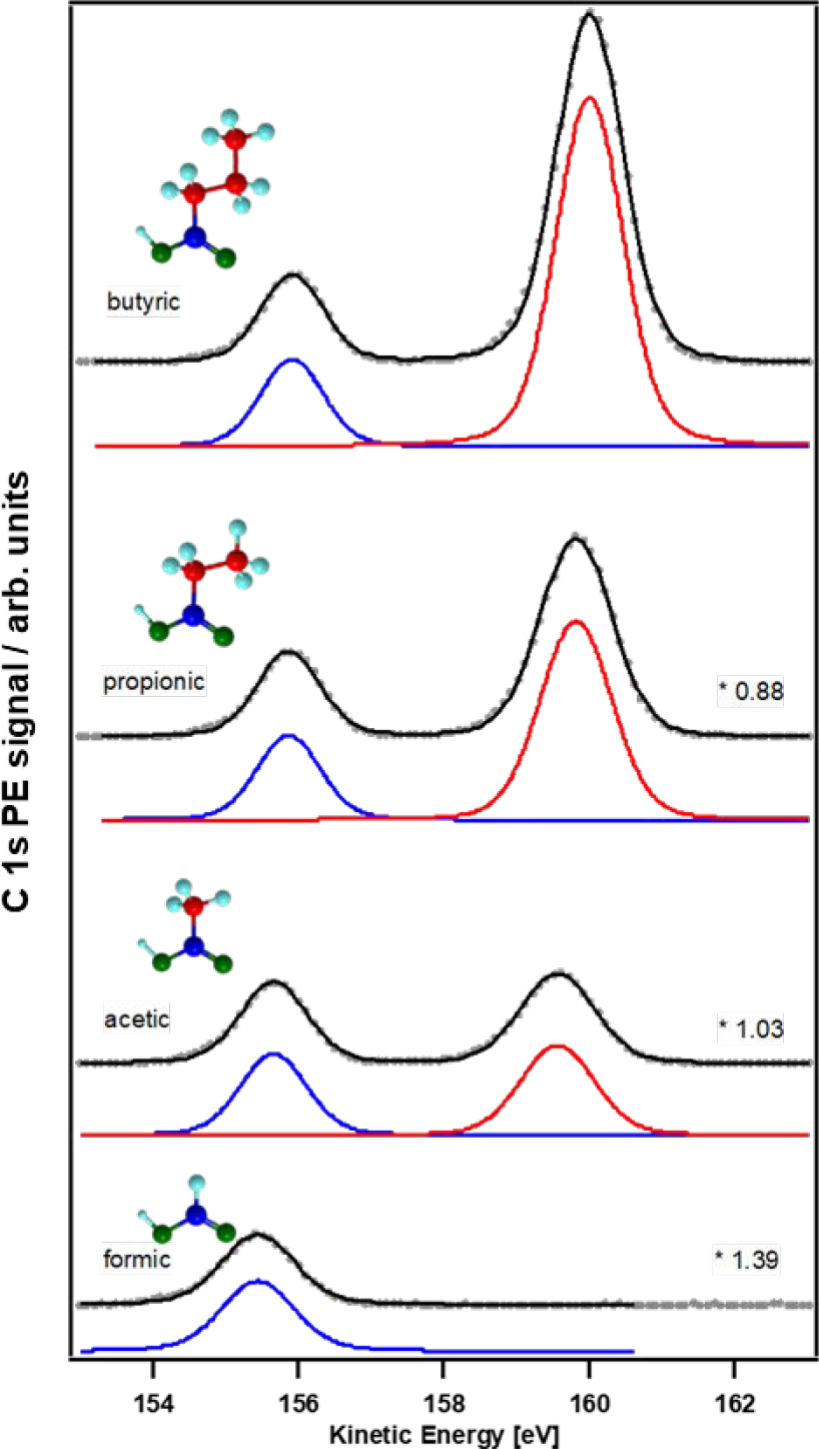
XPS spectra of 0.5 M solutions of formic, acetic, propionic, and butyric acids, with the lower KE (higher BE) peak corresponding to the protonated carboxylic acid group and the higher KE (lower BE) peak corresponding to the aliphatic carbons. The spectra are scaled to display comparable carboxyl-group-related C 1s intensity. Reproduced with permission from ref (1). Copyright 2016 American Chemical Society.

An important benchmark for XPS has been to compare the surface composition derived from XPS with macroscopic surface tension measurements, which is the classical standard technique for assessing the surface excess of solutes.^1^Figure 1 shows a series of C 1s photoemission spectra from 0.5 M solutions of C1 to C4 monocarboxylic acids with the carboxyl group in position 1. They show a clear chemical shift between the carboxyl group carbon and the aliphatic carbon, with the former appearing at lower KE and thus higher BE due to its more oxidized form. The ratio of the two peaks corresponds approximately to the stoichiometric ratio. The spectra are scaled to provide similar carboxyl group carbon C 1s heights; the scaling factors indicated in the figure then directly show, for instance, that the signal for formic acid is lower than that for propionic acid due to the higher surface activity of the latter.

**Figure 2:**
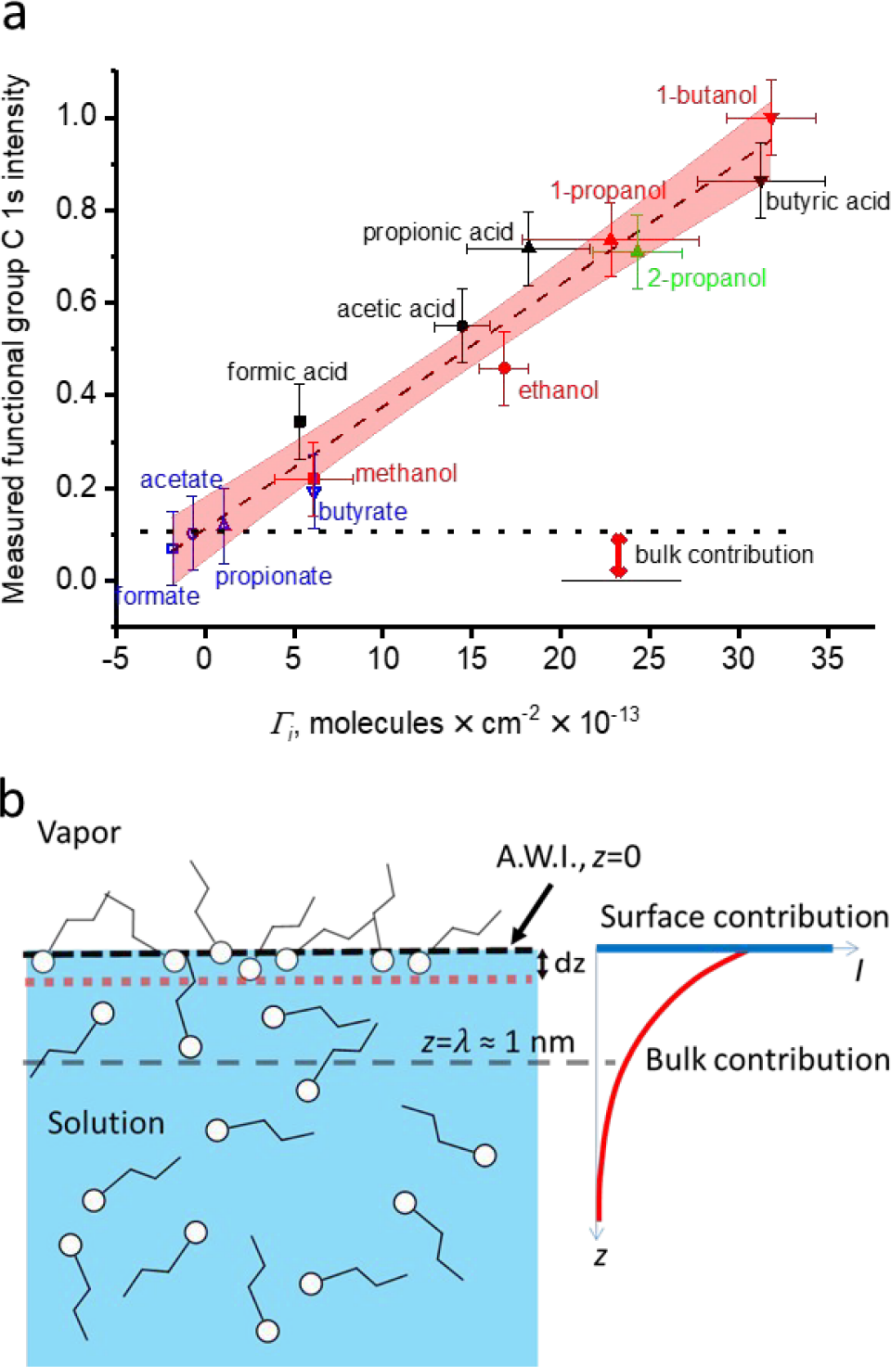
(a) Measured functional group C 1s intensity of organics versus the surface excess derived from surface tension data as a function of organic species (red, alcohols; black, carboxylic acid; blue, sodium carboxylates; squares, C1; circles, C2; upward triangles, C3; and downward triangles, C4). The dashed line represents a linear fit through the data, with the red area representing the 95% confidence intervals. The horizontal dotted line indicates the intersection of the linear fit line with the C 1s intensity scale at zero surface excess, interpreted as the bulk contribution to the C 1s intensity. (b) Conceptual model of how bulk and surface molecules contribute to the signal intensity. A.W.I. indicates the air (vapor)–water–interface. Reproduced with permission from ref (1). Copyright 2016 American Chemical Society.

Figure 2a shows the C 1s photoemission intensity of the functional group carbon (thus one per molecule) for 0.5 M solutions of C1 to C4 monofunctional alcohols, carboxylic acids (at pH < p*K*_a_), and carboxylates (at pH > p*K*_a_). Since the photoemission signal is proportional to the density of the corresponding atom within the probing depth, it is expected to be directly proportional to the surface density of organic molecules. Indeed, this signal is linearly correlated with the surface excess derived from the measured surface tension data provided in the literature. In more detail, since the probe depth is greater than the width to which the hydrated functional groups are confined, the C 1s signal also has a contribution from the bulk of the solution, where the solute density is the same for all solutes, 0.5 M, manifested as a constant offset. In this simple model depicted in Figure 2b, the signal contribution from the surface species is assumed not to be attenuated, while the contribution from the solutes in the bulk decreases exponentially with depth. Thus, while XPS clearly remains sensitive to the near-surface region of the topmost few nanometers,^41^ the sensitivity to the surface is also related to the relative abundance in the bulk and at the surface.

**Figure 3:**
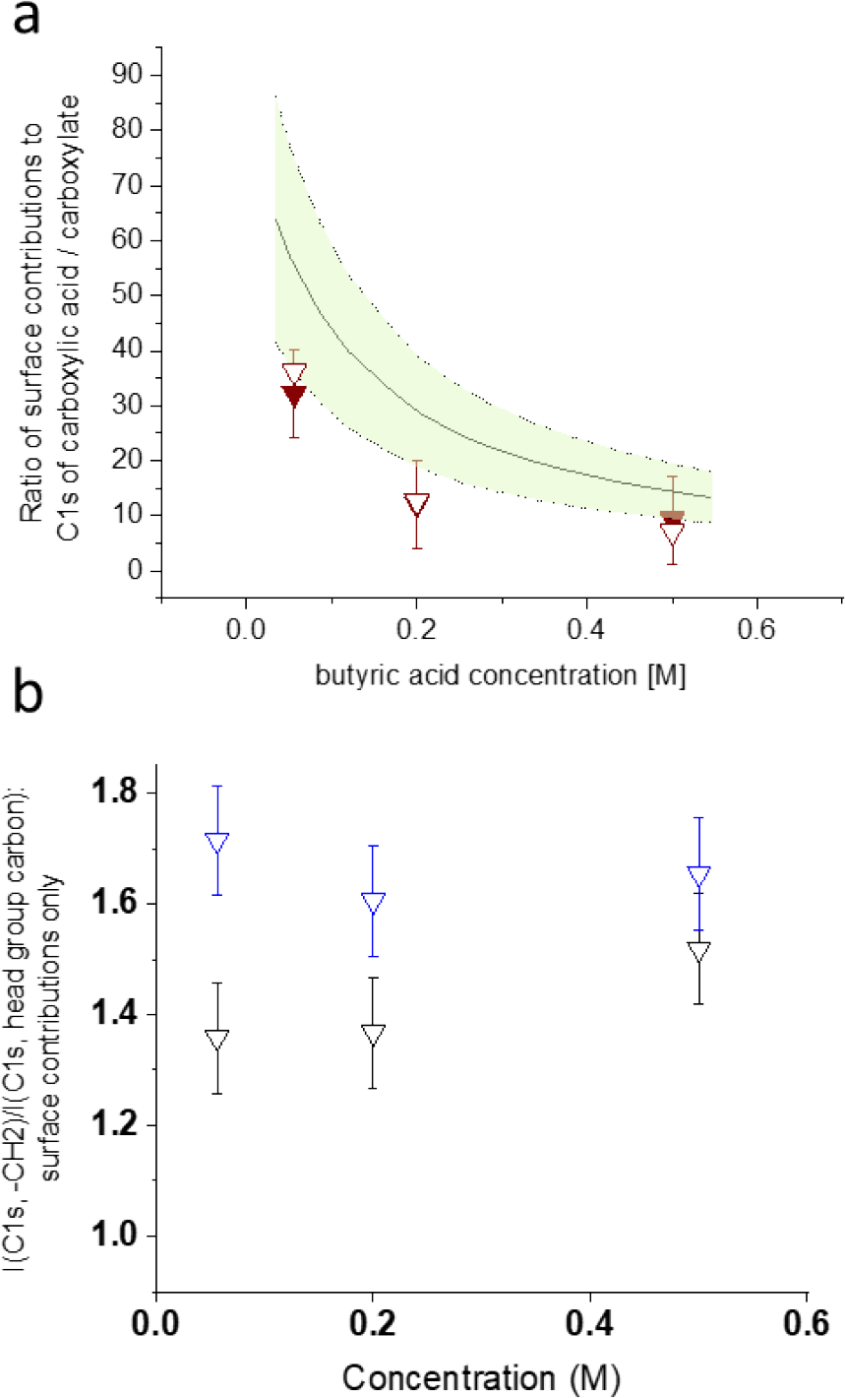
(a) Ratio of the surface contributions to the C 1s signals for butyric acid and butyrate after subtracting the bulk contribution, as a function of the bulk solute concentration, based on the functional group C 1s intensity (filled symbols) and aliphatic chain C 1s intensity (open symbols). The solid line depicts the ratio of the corresponding surface excesses with the shaded area giving the 95% confidence interval. (b) Ratio of aliphatic carbon to headgroup carbon C 1s signal intensity ratio for butyric acid (black) and sodium butyrate (blue) solutions. Reproduced with permission from ref (1). Copyright 2016 American Chemical Society.

As obvious from the data in Figure 2, the carboxylate series is at lower surface excess and correspondingly lower signal intensity than the corresponding acids. The upper panel of Figure 3 demonstrates more than a factor of 30 between the surface contribution to the C 1s headgroup intensity for butyric acid and that for butyrate.^1^ The solid line in the figure is the ratio calculated from the surface excess data obtained from the same surface tension data as used in Figure 2. The ratios measured by photoemission remain somewhat below those, attributed to the fact that the carboxyl group C 1s intensity was not corrected for attenuation by the aliphatic carbons eventually residing above the interface, which leads to an underestimation of the number of surface species. The surface activity of both the neutral carboxylic acid and the charged conjugate base is related to the headgroup attempting structure-breaking hydrophilic solvation, while the aliphatic portion of the molecule requires free-energy-expensive structure-making hydrophobic solvation. The large difference in surface propensity between butyric acid and butyrate is a consequence of the negative charge, pulling the anion away from the interface into the solution as much as possible due to the classical repulsion of ions from the interface between a dielectric medium and air.^42^ This is directly apparent also for the formate ion, which now has an aliphatic portion. It exhibits negative surface excess at the same 0.5 M concentration and features the lowest measured C 1s signal intensity within the series (Figure 2). The picture of the butyrate ions being pulled more toward the bulk of the solution than the neutral butyric acid molecules is manifested in a stronger upward orientation of the aliphatic backbone. This is shown in the bottom panel of Figure 3, where the aliphatic carbon C 1s to headgroup carbon C 1s signal intensity ratio is plotted as a function of the solute concentration. If the molecules have a net upward orientation, then the photoelectrons from the aliphatic carbons are less attenuated than those from the headgroup carbons, leading to ratios >1 (after accounting for the stoichiometry).^43^ Obviously, this ratio is higher for butyrate than for butyric acid.

Other studies also confirmed the higher surface propensity of the neutral acid over its conjugate ion for other organic acids^44^ and also for inorganic acids HNO_3_ and H_2_SO_4_.^45^ Conclusions about the acidity of the interface should be considered with care because the interfacial concentration of H^+^ or H_3_O^+^ is not determined.

## Surface Propensity of a Neutral Alkylamine Base

**Figure 4:**
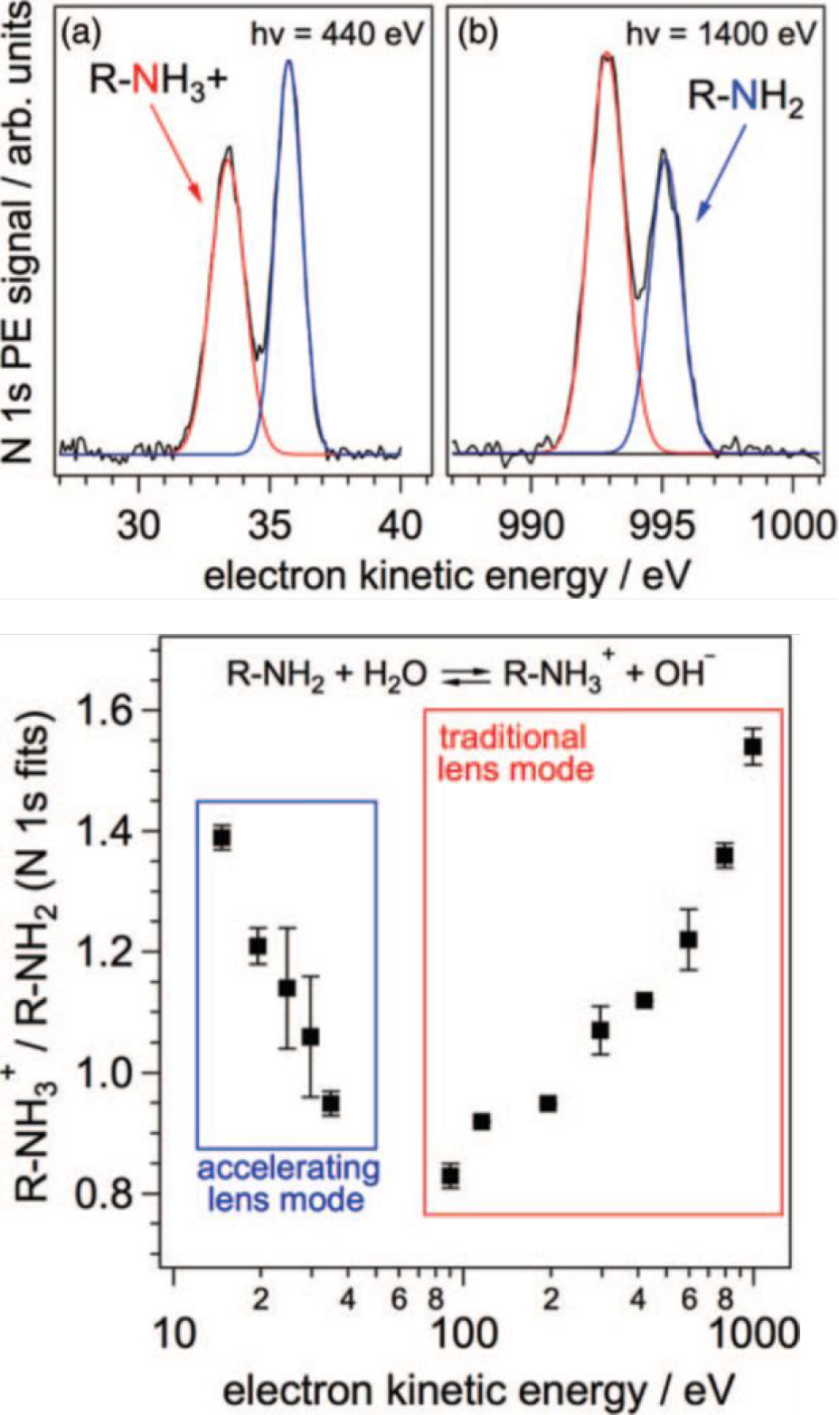
(Top) N 1s photoemission spectrum of 0.25 M butylamine at a pH of 10.6 at photon energies of (a) 1400 and (b) 440 eV. The red and blue lines represent fits of the protonated and neutral amine components, respectively. (Bottom) Ratio of the N 1s photoemission intensity components of butylammonium to those of butylamine as a function of the kinetic energy. Reproduced with permission from ref (39). Copyright 2013 AIP Publishing LLC.

To illustrate the importance of the electrostatic interactions for the surface propensity in the case of bases, we consider an amine. Figure 4a,b shows examples of N 1s spectra from a 0.25 M butylamine solution at pH 10.6, equal to the p*K*_a_ of butylammonium.^39^ The N 1s spectra feature the clearly resolved contributions from butylammonium and butylamine, with the protonated amine at lower KE (higher BE). Clearly, their intensity ratio (bottom panel in Figure 4) depends on the KE and follows roughly the shape of the KE dependence of the inelastic mean free path (IMFP) in water,^34a^ thus it increases with probing depth above 100 eV. The mode of the electrostatic lens system used to get the low KE data^39^ and the magnitude of the IMFP at low KE^46^ are not discussed here. The amine (the base, neutral) is more abundant at the interface than the conjugate ammonium (the acid, charged). While the different behavior of carboxylic acids and bases can be understood in terms of shifted acid–base equilibria,^47^ we emphasize here the role of electrostatic interactions.

## Interfacial Abundance and Reactivity of Bromine Species

**Figure 5:**
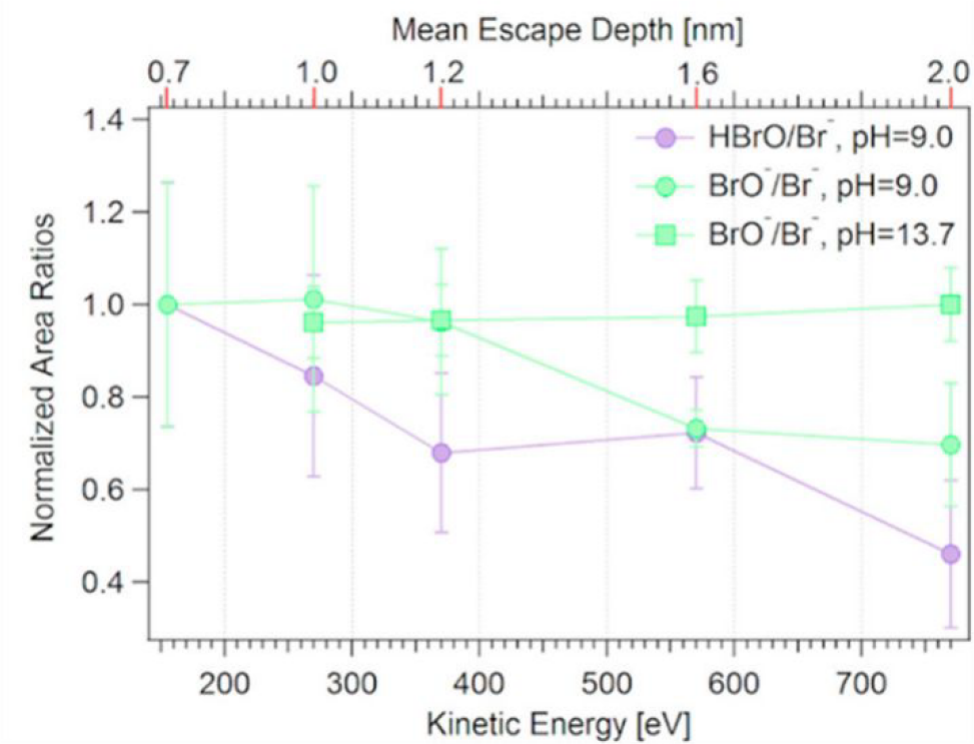
(Left) BrO^–^ and HOBr to bromide Br 3d photoemission intensity ratio as a function of the photoelectron kinetic energy at pH 9 for HOBr and pH 13 for BrO^–^. Reproduced with permission from ref (2). Copyright 2020 American Chemical Society.

The classical electrostatic repulsion^42^ can qualitatively explain the contrasting surface propensity for acid–base pairs. Large polarizable ions, such as those of the halide series, had been suggested to prefer the asymmetric environment at the interface.^48^ Surface tension measurements indeed indicate a less strongly increasing surface tension with solute activity and thus less depletion for the more polarizable iodide than for fluoride. The abundance of halide ions and their oxidation are very important in atmospheric chemistry as mentioned upfront.^12b^ Classical molecular dynamics simulations with polarizable force fields supported the idea of iodide and bromide being even enhanced at the aqueous solution–air interface.^48^ This was first confirmed by XPS experiments on static, highly concentrated solution droplets.^49^ In addition, kinetic experiments suggested that the oxidation of halide ions by OH radicals and ozone was accelerated at the surface, attributed to such halide enhancement.^50^ However, more recent liquid microjet XPS experiments showed a significantly lower abundance of iodide and bromide at the interface, which was also supported by new ab initio molecular dynamics simulations.^2,34b,51^ Similar behavior was also observed for more oxidized bromine species, BrO^–^ and BrO_3_^–^.^2^ In turn, as for other acid–base pairs discussed above, the conjugate acid of the former, neutral HOBr is indeed surface-active (Figure 5).

**Figure 6:**
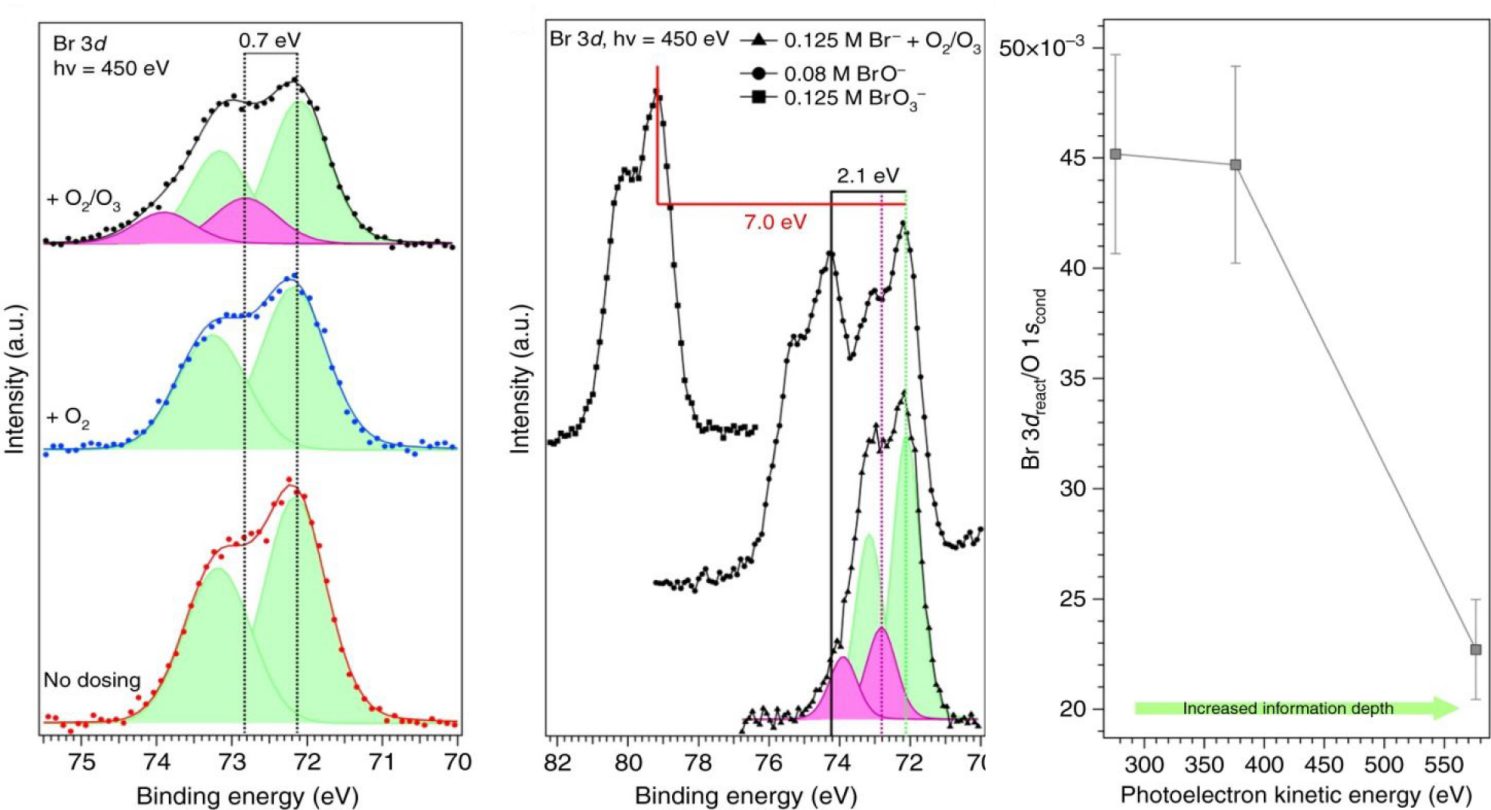
(Left) Br 3d photoemission spectra of the Br 3d peak acquired in vacuum (bottom), in 0.1 mbar O_2_ (middle), and with a few % of O_2_ converted to O_3_ (still at 0.1 mbar total pressure), demonstrating the formation of the bromide ozonide intermediate. (Middle) Comparison of the bromide ozonide intermediate spectrum (bottom) with reference solutions containing hypobromite (middle) and bromate (top). (Right) Bromide ozonide Br 3d to O 1s photoemission signal intensity ratio as a function of photoelectron kinetic energy demonstrating the surface propensity of the intermediate. Reproduced from Artiglia et al. Adapted with permission from ref (3). Copyright 2017 The Authors, some rights reserved; exclusive licensee Springer Nature. Distributed under a Creative Commons Attribution 4.0 Unported (CC BY 4.0) License.

The surface activity of HOBr (the direct oxidation product of bromide) could have been one of the reasons that the formation of Br_2_ at the interface from the oxidation of bromide by ozone was observed to be enhanced, as HOBr reacts with Br^–^ to yield Br_2_.^50a^ However, the involvement of a surface process was already apparent from the ozone loss kinetics, the first step in bromide oxidation.^50a^ For this reaction, a bromide ozonide complex was suggested as an intermediate in the bulk aqueous phase.^52^ Liquid microjet XPS could identify this intermediate and demonstrate its strong preference for the liquid interface (Figure 6).^3^ The identification was supported by BE calculations, and the surface preference, by molecular dynamics simulations.^3^ The surface propensity is apparent from the strongly decreasing ratio of the Br 3d signal to the O 1s signal of liquid water with increasing KE and thus probing depth. We note that we did not observe the BrO^–^ product, as the overall kinetics are too slow for the short time scale of the interaction. The gas–surface reaction time scale of a few hundred microseconds is long enough for this non-surface-active ion to diffuse beyond the probe depth of XPS. This example emphasizes that a significant surface coverage of at least 10^12^ molecules cm^–2^ (percent of a monolayer) and significant surface propensity are required for the successful observation of the intermediate. The observation is therefore constrained by the steady state established among the rate of production of the species of interest at the surface, its rate of solvation, and the rate of diffusion toward the bulk underneath to attain bulk–surface equilibrium.

## Surfaces of Fe(II)- and Fe(III)-Containing Aqueous Solutions

**Figure 7:**
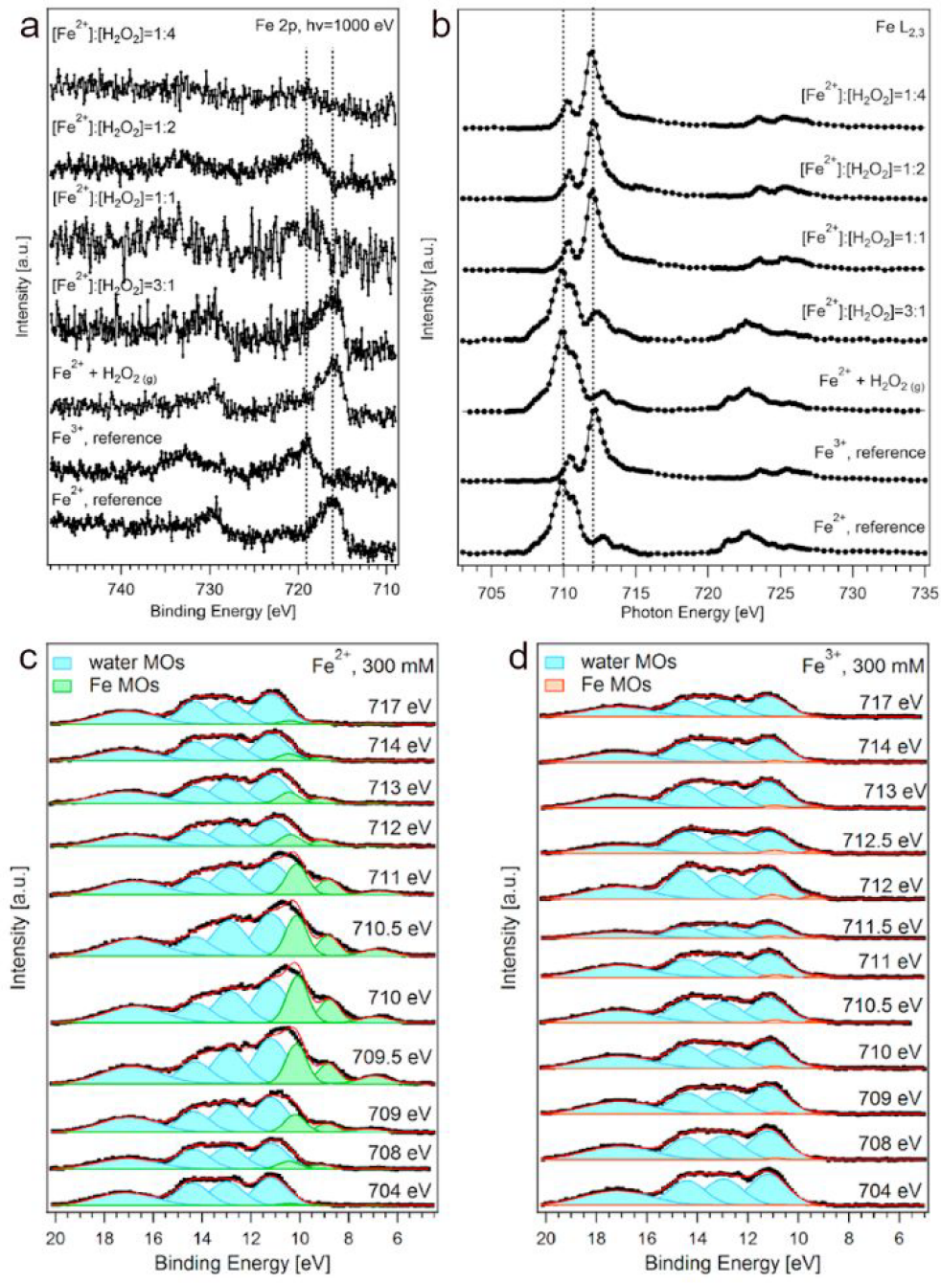
(a) Fe 2p photoemission spectra and (b) NEXAFS spectra of the Fe L_2,3_ edge of Fe^2+^ and Fe^3+^ 300 mM aqueous solutions, which are homogeneous Fenton reagents having different [Fe^2+^]/[H_2_O_2_] and gaseous H_2_O_2_ dosed around a 300 mM Fe^2+^ solution. (c, d) RPE of Fe^2+^ and Fe^3+^ 300 mM aqueous solutions, respectively. Adapted with permission from ref (4). Copyright 2022 American Chemical Society.

As an example of a completely different type of aqueous solution, we have investigated the local coordination environment of ferrous (Fe^2+^) and ferric (Fe^3+^) ions in aqueous solutions and the reaction of Fe^2+^ with hydrogen peroxide, which is well known as Fenton’s reaction.^4^ The latter is key in the role of iron in the environment.^16,18,20,53^ It has been shown that high-valent iron species (ferryls) form preferentially at the gas–liquid interface upon interaction with gaseous H_2_O_2_, tentatively assigned to an enhanced lability and/or distorted geometry of the Fe^2+^ hydration shell.^54^ We performed XPS measurements on a liquid microjet to investigate the electronic state and local environment of Fe^2+^ and Fe^3+^ aqueous solutions (Figure 7a). The iron to oxygen atomic ratio derived from the Fe 2p and O 1s photoemission signals was below that expected for the bulk concentration for both Fe^2+^ and Fe^3+^ within the topmost nanometer, consistent with repulsion due to their significant charge.^55^ A more detailed analysis of the Fe 2p core levels, accompanied by partial electron yield near-edge X-ray absorption fine structure (NEXAFS) spectra at the Fe L_2,3_ edge (Figure 7b) and resonant photoemission (RPE) of the valence band across the Fe L_3_ edge (Figure 7c,d), shows that Fe^2+^ and Fe^3+^ form octahedral high-spin complexes. In particular, RPE allows identifying the splitting and population of the 3d orbitals of iron ions. Figure 7c,d shows that the intensity of the d orbitals of Fe^2+^ and Fe^3+^, highlighted in green and orange, respectively, is amplified when scanning the photon energy across the L_3_ edge of iron (i.e., from 704 to 717 eV). Fe^3+^ adopts the expected configuration of a high-spin complex, with t_2g_ and e_g_ orbitals separated by the crystal field splitting parameter. On the contrary, Fe^2+^ has a spin-unrestricted crystal field, which suggests a deviation from the ideal octahedral symmetry.^29b^ Theoretical calculations supported the rapid formation of complete octahedral complexes and the deviation of [Fe(H_2_O)_6_]^2+^ from the ideal symmetry.^4^ Next, we addressed the reaction of Fe^2+^ with H_2_O_2_, the key reaction of Fenton chemistry. We first looked at premixed solutions with varying [Fe^2+^]/[H_2_O_2_] molar ratios (mixtures prepared prior to injection). Both XPS and NEXAFS data indicated the quantitative conversion of Fe^2+^ to Fe^3+^, as expected based on the kinetics (Figure 7a,b). Furthermore, we dosed gaseous H_2_O_2_ (10^–4^ mbar partial pressure) in situ around the liquid filament containing Fe^2+^. As in the case of O_3_ reacting with bromide discussed above, for the few hundred microseconds of interaction time we do not expect to see significant amounts of Fe^3+^, given the rapid diffusional exchange and the absence of surface activity for both Fe^2+^ and Fe^3+^ complexes. No additional iron species at the surface, such as a ferryl, which would likely appear at different BEs or as a separate feature in the NEXAFS spectra, could be detected in the premixed solutions or in the case of in situ dosing of gaseous H_2_O_2_ (Figure 7a,b). To summarize, the combined experimental and theoretical results suggest that ferrous ions lack surface propensity and their aquo complexes adopt a slightly distorted but complete octahedral configuration. In turn, no ferryls were detected while dosing 10^–4^ mbar of gaseous H_2_O_2_ around a liquid filament containing a 300 mM Fe^2+^ aqueous solution. While the spectroscopic techniques adopted in this work are powerful and reliable tools for identifying the structure and arrangement of iron ion complexes in the interfacial region of aqueous solutions, the experimental conditions might not be in the right regime to allow the detection of the previously identified surface intermediates in the reaction with gaseous H_2_O_2_.^56^

## Impact of Surfactants on the Surface Abundance and Reactivity of Bromide

**Figure 8:**
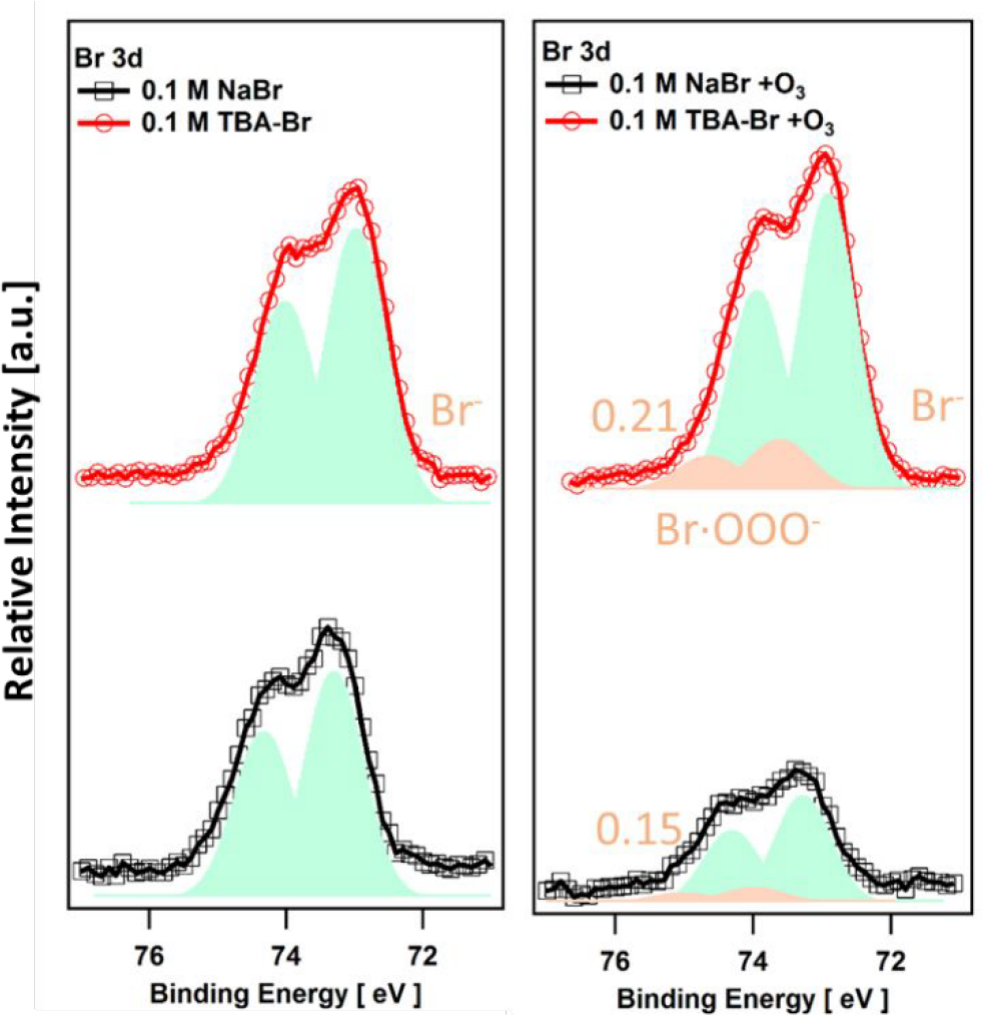
Br 3d photoelectron spectra of 0.1 M TBA-Br and 0.1 M NaBr aqueous solutions at a photon energy of 450 eV in the absence (left) and presence (right) of O_3_ and in the absence (bottom) and presence (top) of TBA. The spectra within each panel share the *y*-axis scale. Adapted with permission from ref (2). Copyright 2016 American Chemical Society.

Increasing the degree of complexity, we next address how organic species affect the oxidation chemistry of halide ions. Surface-active organic compounds are present at the ocean surface and are associated with sea spray aerosol.^10^ We investigated the impact of a cationic surfactant, tetrabutylammonium (TBA), on the abundance and reactivity of bromide. The results show that its association with TBA leads to much higher bromide abundance at the interface than in the absence of a surfactant (Figure 8).^57^ The difference in the Br 3d signal does not appear to be very large. However, we need to take into account the fact that TBA leads to a layer on top of the solution, which attenuates photoelectrons originating from the aqueous phase below. The true interfacial concentration of bromide was quantified by using an attenuation model and an assumed structure of the interface, based on previous work.^58^ A parallel increase in the interfacial concentration of the bromide ozonide intermediate has been observed in the presence of O_3_. In separate kinetics experiments, the reaction rate of ozone was also observed to increase in comparison to that of neat bromide solution. The bromide and ozonide intermediate signals could be consistently explained using the attenuation model, which then also provided insights into the subtle effects of how this surfactant affected the reactivity.^57^ In turn, the nitrogen N 1s (not shown here) signal indicated that the detailed structure of the TBA at the interface might be more complex than a simple well-organized monolayer. He backscattering experiments,^59^ providing higher depth resolution, have indicated a larger interfacial thickness for a similar system. Thus, the example discussed here clearly demonstrates how the electrostatic interactions of an ionic surfactant lead to changes in both abundance and reactivity. In a less dramatic way, neutral carboxylic acids or alcohols also feature effects on the distribution of anions, such as bromide or iodide, as we have demonstrated previously with butanol,^58,60^ butyric acid,^58^ and citric acid.^61^

## Conclusions and Outlook

The cases summarized above reveal the potential to broaden this research toward other major inorganic and organic solutes and their mixtures. For the relatively simple aqueous solutions considered, the interface is fairly simple in structure, with the transition from gas to surface to bulk occurring within ∼1 nm.^42b^ Thus, the interpretation of the signals has been relatively straightforward. In turn, for more complex systems with multiple ions of different charge and surfactant layers of complex structure, the interface may span over larger depths and the differentiation among the surface, interfacial region, and bulk is not straightforward anymore. XPS experiments could involve angle-dependent measurements^41a^ or complementary molecular beam scattering experiments.^59^ Nonlinear optical spectroscopy^62^ could also be used to constrain such cases further.

An important aspect is the solute concentration in atmospheric aqueous aerosol particles. It is controlled by water activity, thus ambient relative humidity and temperature, meaning extremely high molarities and often supersaturated solutions. Under such conditions, many chemical reactions operate in completely different regimes. For instance, the reaction of nitrogen dioxide (NO_2_) with sulfite, which is well established in dilute solution, is suspected to operate through a surface-specific intermediate on realistically concentrated aerosol particles.^63^ Another aspect is that especially organic-rich particles tend to form highly viscous phases exhibiting drastically different chemical properties that are relevant to climate and health impacts.^20^ A substantial gap remains between the presently achievable solution concentrations in a liquid microjet, driven by the need to avoid salt precipitation at the point of dispatching the liquid into the vacuum, and those pertaining to atmospheric aerosol particles. While the operation of the liquid jet at equilibrated water vapor pressure would help in terms of the physical properties, XPS in the millibar range would provide much less spectroscopic detail.^40^ Another option is to form a droplet train to allow particles to evaporate and equilibrate at low water vapor pressure and attain such highly supersaturated solution states.^64^ An upgraded version of such an experiment is currently in development.^65^ Many open questions are related to the hydration of ions in the bulk and on the surface of such concentrated solutions because hydration and ion pairing strongly influence many processes. Advanced spectroscopic techniques such as RPE, or Auger electron spectroscopy, considering resonant effects as well as intermolecular interactions, provide direct information on nearest neighbors, hydration shells, and ion pairs^29b,66^ and could be exploited for such systems.

Another aspect is the water structure itself. Auger electron yield NEXAFS spectroscopy provides a surface-sensitive tool for probing the local hydrogen bonding structure in aqueous solutions.^29a,67^ We have recently started to explore this technique to reveal water ordering underneath monolayers of phenolic species at the water surface.^68^ As indicated above, the Auger process involves multiple decay processes, each of which carries specific information. Keeping in mind that the Auger electron yield NEXAFS spectra contain integrated data, more details would become available by performing resonant Auger spectroscopy at the O K-edge and using the details of that to establish a link to water ordering.^66c^ These would then also allow direct comparison to the structure-sensitive nonlinear optical spectroscopy methods.^27^ It would also represent a significant advance to combine the probes discussed here with X-ray emission mode experiments (e.g., resonant inelastic X-ray scattering that probes high vibrational levels of liquid water^69^ but remains less selective for the surface).

On the technical side, the liquid microjet environment also offers other configurations, for instance, in the design of the injection nozzles. With the advent of microfluidic devices, such nozzles could be flexibly engineered to combine the mixing of different precursor liquids to offer access to short reaction time scales, with the possibility to dose gases.^70^ More slowly reacting systems (seconds to minutes time scales) can also be addressed by premixing liquids to obtain adjustable reaction times prior to injection before the XPS measurement.

We have not touched on the fact that atmospheric particles are often internal mixtures of multiple phases, such as featuring several liquid phases^71^ or a liquid surrounding solid inclusions (e.g., the nonsoluble mineral fraction). Nanoparticle suspensions in aqueous solution indeed offer an opportunity for liquid jet XPS, which has not yet been exploited for atmospherically relevant systems.^72^

Finally, we have focused our review on the experimental aspects and the experimental results. As indicated multiple times in the discussion, interpretation often profits from theory support. As molecular dynamics and electronic structure calculations are now able to address larger molecular systems, combined theoretical and experimental investigations are increasingly important.^2,4,73^
